# The piecewise parabolic method for Riemann problems in nonlinear elasticity

**DOI:** 10.1038/s41598-017-13484-z

**Published:** 2017-10-18

**Authors:** Wei Zhang, Tao Wang, Jing-Song Bai, Ping Li, Zhen-Hua Wan, De-Jun Sun

**Affiliations:** 1 Department of Modern Mechanics, University of Science and Technology of China, Hefei, 230027 China; 2 School of Civil Engineering and Architecture, Southwest University of Science and Technology, Mianyang, 621010 China; 3 Institute of Fluid Physics, China Academy of Engineering Physics, Mianyang, 621900 China

## Abstract

We present the application of Harten-Lax-van Leer (HLL)-type solvers on Riemann problems in nonlinear elasticity which undergoes high-load conditions. In particular, the HLLD (“D” denotes Discontinuities) Riemann solver is proved to have better robustness and efficiency for resolving complex nonlinear wave structures compared with the HLL and HLLC (“C” denotes Contact) solvers, especially in the shock-tube problem including more than five waves. Also, Godunov finite volume scheme is extended to higher order of accuracy by means of piecewise parabolic method (PPM), which could be used with HLL-type solvers and employed to construct the fluxes. Moreover, in the case of multi material components, level set algorithm is applied to track the interface between different materials, while the interaction of interfaces is realized through HLLD Riemann solver combined with modified ghost method. As seen from the results of both the solid/solid “stick” problem with the same material at the two sides of contact interface and the solid/solid “slip” problem with different materials at the two sides, this scheme composed of HLLD solver, PPM and level set algorithm can capture the material interface effectively and suppress spurious oscillations therein significantly.

## Introduction

Nonlinear elastic deformation of solid material undergoing high-load conditions commonly occurs in industrial application areas, such as the design of automobile anti-collision device, the evaluation of the capability of spacecraft structural materials against hypervelocity impact, and etc. For such a phenomena of technological interest, the research strategy can be classified into three categories: Lagrangian view^1,2^, Eulerian view^3–5^ and the combination of the above two views^6^. The Lagrangian view is more popular in engineering area as the mesh point for this view is always coincident with material point in the deformation process of material and no material point crosses the mesh boundary, leading to no necessity for processing material interface. While the material has a large deformation, the computational mesh will warp or distort. Although this problem can be overcome through mesh remeshing and deleting the abnormal meshes, the effects on the computational accuracy cannot be ignored yet. While for Eulerian view, fixed meshes are adopted and materials are allowed to pass through mesh interface, implying that mesh warp or distortion induced in the case of large deformation could be avoided in a large extent. Thus, it is relatively suitable for studying the problems featured with large material deformation. Despite of this, it is still a great challenge to apply the Eulerian reference frame as the capturing of the interfaces between different materials is quite complicated therein.

Elastic deformation of solid material is a common phenomenon in engineering, but to describe large deformation process^7^ accurately is still a tough task. In recent years, a variety of model equations and numerical methods have been developed under Eulerian view to simulate nonlinear elastic deformation behaviors of solid material, as reviewed by Benson^8^, Wilkins^9^. With regard to model equation, the ones of elastic strain law of materials should be added in Eulerian reference frame on the basis of the governing equations in Lagrangian reference frame, which are composed of mass, momentum and energy equations. The model equations describing elastic strain law can be divided into two major groups. The first one, whose variable is deviator stress tensor^10–12^, is constructed in non-conservative form and thermodynamically inconsistent. It is extensively applied in engineering as it could also account for plastic effect of solid material through Maxwell relaxation model, despite that its disadvantages of not ensuring the strict numerical resolution due to the non-conservative form and being unable to treat strong variables (shock, expansion wave, etc.)^4^ correctly, are also obvious. With this in mind, several authors have put forward another group of model equation, which is in conservative form and based on deformation tensor. This group of model equation can also be classified into two types. The first type proposed by Romenski^13^ and Godunov^14^ contains 9 transport equations for the components of deformation gradient tensor (*F* = ∂*x*/∂*X*, defined as the gradient of Eulerian coordinates to Lagrangian coordinates). Effects of plastic deformation are considered by adding source terms into these transport equations. The second one is constructed on the basis of inverse deformation gradient tensor (*f* = *F* ^−1^ = ∂*X*/∂*x*, namely gradient of Lagrangian coordinates to Eulerian coordinates). This model, which needs to solve 21 equations in three dimensions, was firstly suggested by Plohr & Sharp^3^ for elastic solids and then developed to be applicable to rate-dependent and rate-independent plasticities. It is worthwhile that the two types of strain law equations based on deformation gradient tensor and inverse deformation gradient tensor are mathematically equivalent.

Under Eulerian view, numerical methods for the simulation of nonlinear elastic deformation behavior need obviously to be able to handle large deformation simultaneously with high resolution, considering the potential complex mechanical behavior. The Godunov scheme based on the solution of local Riemann problem, which is an efficient method for shock capturing in fluid mechanics (see Toro^15^), satisfies this requirement and can be thus seen a reasonable choice for the simulation of deformation behavior. In 1959, Godunov proposed the original form of well-known Godunov scheme with only first-order accuracy in both space and time, which meant to solve exact Riemann problem at each inter-cell boundary, for a hyperbolic equation system of conservation laws^16,17^. Hereafter, numerous works are devoted to the improvement of the Godunov scheme, including increasing the precision and resolution of the scheme as well as decreasing the computational time by using the approximate solution of Riemann problem to replace the exact solution (see Garaizar^18^, Miller^19^, Titarev^20^ and Barton^21^). For the former, several researchers derived the scheme with second-order accuracy in space (e.g. MUSCL) by modifying the constant approximation of original variables to linear distribution^4,22^; while for the latter, Titarev^20^ discussed several approximate solution methods for nonlinear elasticity, including GMUSTA and EVILIN solvers, linearized method, and FORCE flux method. The other approximate methods include linearized method of Miller^22,23^, HLLC solver of Gavrilyuk^4^, Yannick Gorsse^24^, Alexia de Brauer^25^, Gavrilyuk^4^, HLLEM solver of Dumbser^26^ and HLLD solver of Lopez Ortega^27^ which is often used in magnetohydrodynamics(MHD) and is rarely utilized in solid mechanics. The results of these approximate methods in elasticity problems imply that these methods could obtain considerably accurate solutions except in some special cases where the lack of the robustness of linearized method may lead to the failure of solution process or the HLL and HLLC solvers are incapable of capturing the seven-wave structure in solid materials precisely.

Considering the case of multi-component solid material system, it is necessary to select proper numerical method for capturing the material interfaces and therewith solving the interaction between different materials. Under Eulerian’s frame, the methods of interface tracking type, such as level-set method, volume of fluid (VOF) method and marking particle method, are proved to be suitable for the treatment of multi-component solid material problems, where it is necessary to keep the sharp shape of interface and interface smearing is unacceptable. The successful applications of these methods with solid mechanics model of conservative form include the works by Miller^22^, Wang^28^, Walter^29^ and Barton^30^. It is worthwhile that the over shoot phenomena, i.e. the non-physical oscillation at material interfaces, often occur when common numerical methods are used with interface tracking method. Thus, the highly efficient and reliable numerical method is needed to be developed with interface tracking method to achieve the two objects of studying the interaction between different materials and decreasing numerical oscillation at material interfaces at the same time.

In this study, we develop Godunov scheme for solving Riemann problems in nonlinear elasticity based on the coupling model developed by Godunov & Romenski^14,31^ in the Eulerian reference frame. On one hand, the capabilities of HLL family of Riemann solvers in the single elastic material problem are evaluated and compared. On the other hand, the Piecewise Parabolic Method (PPM)^32^, which is widely applied in the field of fluid mechanics^23,33^, is employed to achieve higher-order spatial accuracy for simulating the mechanical behaviors of materials under the conditions of high-pressure and high strain rate. Furthermore, the efficiencies of these improvements as well as the usage of level set method for interface tracking in problems with multi material components are all examined in shock-tube problems, solid/solid “stick” problem, solid/solid “slip” multi-material problem, etc.

## Results

In this section, we first present a comparative study of the HLL-type Riemann solvers which are applied in single material cases. (Particularly, we select test cases that are known to cause severe difficulties for numerical computations to assess the accuracy and robustness of the schemes.) With the state equation given in Equation (10) employed, Equation (8) is solved in each test case under different initial and boundary condition. The common material parameters, which appear in state equation, are shown in Table 1. Moreover, first-order Godunov’s scheme is extended to PPM, which is featured with higher order of accuracy and used with different types of Riemann solvers for single material problem. Further, the validation of PPM combined with different types of Riemann solvers (especially the HLLD solver) are examined in one or two dimensional cases with multi materials by numerical experiments. In the test cases below, all one-dimensional initial value problems are solved in a computational domain 0 *m* ≤ *x* ≤ 1 *m*, and the initial discontinuity is placed at the location of *x*_0_ = 0.5 *m*. Transmissive boundary conditions are applied at the computational domain boundaries and the CFL number is set to be 0.8 unless otherwise specified.

**Table 1:** The material parameters of equation of state.

Parameters	Cu	Al	Steel	Units
*ρ*_0_	8.93	2.71	8.03	*g*/*cm* ^3^
*c*_0_	4.6	6.22	5.68	*km*/*s*
*c*_*v*_	3.9 × 10^−4^	9.0 × 10^−4^	5.0 × 10^−4^	*KJ*/*gK*
*T*_0_	300	300	300	*K*
*b*_0_	2.1	3.16	3.1	*km*/*s*
*α*	1.0	1.0	0.596	—
*β*	3.0	3.577	2.437	—
*γ*	2.0	2.088	1.563	—

### Test case 1: Contact discontinuity problem

In this case, we solve the Riemann problem with initial density and velocity distributions corresponding to a contact discontinuity. At both sides of this discontinuity, the materials are both copper and the initial distributions are as follows:

$$\begin{array}{c}{W}_{L}:\{u=(\begin{array}{c}0.01\\ 0\\ 0\end{array})\,km/s,F=(\begin{array}{ccc}1.156276139 & {\rm{0.034688284}} & 0\\ {\rm{0.093190648}} & {\rm{1.002195719}} & 0\\ 0 & 0 & 1\end{array}),S={10}^{-3}\,kJ/gK\\ {W}_{R}:\{u=(\begin{array}{c}0.01\\ 0\\ 0\end{array})\,km/s,F=(\begin{array}{ccc}1 & 0.03 & 0\\ 0.02 & 1 & 0\\ 0 & 0 & 1\end{array}),S=0\,kJ/gK\end{array}$$

**Figure 1:**
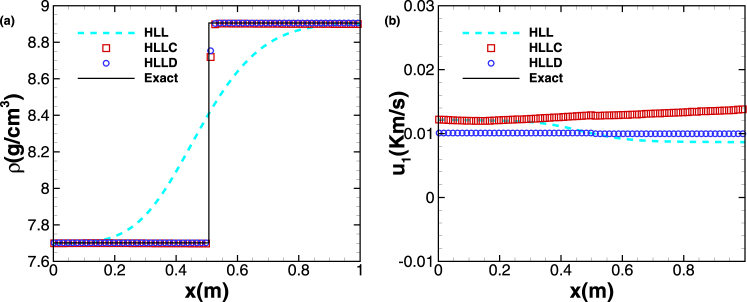
Contact discontinuity problem. The profiles of density (**a**) and velocity (**b**) at *t* = 0.7 *ms*. The symbols are plotted with a spacing of 4 cells. The solid line represents the exact solution.

Here we solve the case within the dimensional time *t* = 0.7 *ms* on a mesh of 500 cells and make a quantitative comparison with the exact solution *W*(*x*, *t*) = *W*(*x* − 0.01*t*, 0). Figure 1 presents the profiles of density *ρ* and velocity component *u* computed by first-order Godunov’s scheme with three kinds of Riemann solvers, i.e. HLL, HLLC and HLLD (In the following we denote the methods of first-order Godunov’s scheme combined with HLL, HLLC and HLLD Riemann solvers by ‘1st + HLL’, ‘1st + HLLC’ and ‘1st + HLLD’, respectively.) It is clearly shown that the HLLC and HLLD solvers produce nearly accurate density profiles which are almost coincident, while the density profile derived by HLL is rather different from the exact solution. For the velocity distribution, the results from three methods, among which only that from HLLD is nearly coincident with the exact solution, are considerably different. The *L*_1_ norm errors and convergence orders of density *ρ* and deformation gradient *F*_11_ at time *t* = 0.7 *ms* for test case 1 are presented in Table 2 for 1st + HLL, 1st + HLLC and 1st + HLLD. It is observed that all first-order methods are converging at the order of $O({\rm{\Delta }}{x}^{\tfrac{1}{2}})$ rather than *O*(Δ*x*).

**Table 2:** *L*_1_ Errors and orders of convergence for the test case 1.

Scheme	N	*ρ*		*F*_11_	
		*L*_1_-error	*L*_1_-order	*L*_1_-error	*L*_1_-order
1st order + HLL	100	2.22E-01	—	2.91E-02	—
	200	1.58E-01	0.491	2.08E-02	0.489
	400	1.12E-01	0.499	1.47E-02	0.497
1st order + HLLC	100	1.09E-02	—	5.25E-03	—
	200	7.66E-03	0.503	3.72E-03	0.498
	400	5.35E-03	0.517	2.59E-03	0.524
1st order + HLLD	100	6.97E-03	—	8.22E-04	—
	200	4.89E-03	0.510	6.17E-04	0.414
	400	3.01E-03	0.701	3.84E-04	0.685

### Test case 2: Five-wave shock-tube problem

In the present case, the materials at both sides of the discontinuity interface still are copper. The initial distributions satisfy compatibility condition and are given as

$$\begin{array}{c}{{\bf{W}}}_{L}:\{{\bf{u}}=(\begin{array}{c}0\\ 1\\ 0\end{array})\,km/s,{\bf{F}}=(\begin{array}{ccc}0.95 & 0 & 0\\ 0.05 & 1 & 0\\ 0 & 0 & 1\end{array}),S={10}^{-3}\,kJ/gK\\ {{\bf{W}}}_{R}:\{{\bf{u}}=(\begin{array}{c}0\\ 0\\ 0\end{array})\,km/s,{\bf{F}}=(\begin{array}{ccc}1 & 0 & 0\\ 0 & 1 & 0\\ 0 & 0 & 1\end{array}),S=0\,kJ/gK\end{array}$$

For this case, the solution structure comprises five waves (from left to right): two left-travelling rarefaction waves, a right-travelling contact discontinuity wave, a right-travelling rarefaction wave and a right-travelling shock wave, respectively. The solution derived by high-order scheme with a fine mesh is used as the reference solution for comparison.

**Figure 2:**
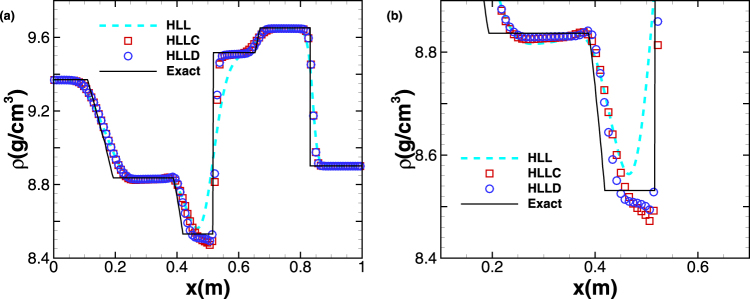
Five-wave shock-tube problem. The profiles of (**a**) density (**b**) partial enlargement at *t* = 0.06 *ms* computed by HLL, HLLC and HLLD.The symbols are plotted with a spacing of 4 cells. The solid line represents the exact solution.

**Figure 3:**
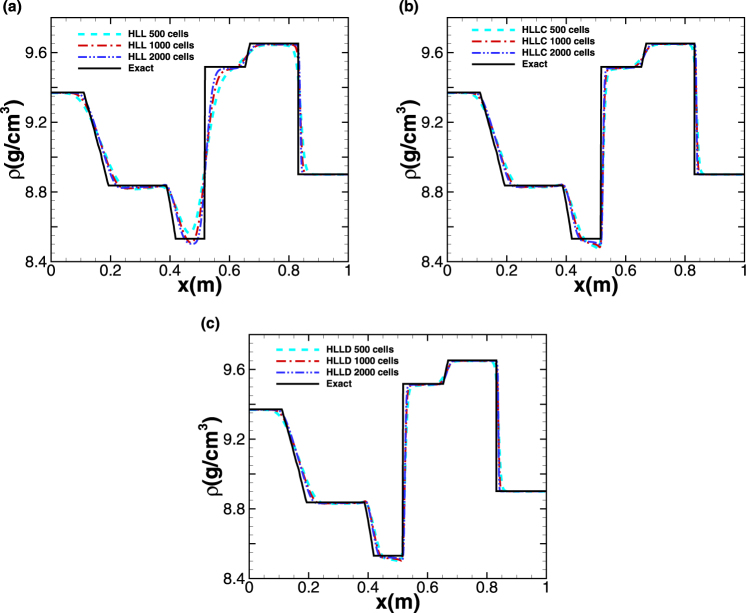
Five-wave shock-tube problem. The profiles of density computed by (**a**) HLL (**b**) HLLC (**c**) HLLD at *t* = 0.06 *ms* with different mesh size. The solid line represents the exact solution.

We solve this problem within the dimensional time *t* = 0.06 *ms* on a mesh of 500 cells by using first order scheme with HLL, HLLC and HLLD solvers. The density profiles are shown in Fig. 2, where the exact solution is denoted by solid line. It is seen that HLL has the most diffusive result in the region 0.4 *m* ≤ *x* ≤ 0.6 *m*, where HLLC and HLLD provide more accurate results. Also, it is found that HLLD performs better than HLLC as shown in the local enlarged graph Fig. 2(b). To check grid convergence, the cell number is increased from 500 to 1000 and 2000 with the same time interval and the results from different gird resolutions are compared in Fig. 3. As shown in Fig. 3(a), the convergence rate is relatively low for HLL, and the density profile with 2000 grid cells is even very diffusive around *x* = 0.5 *m*. With increasing cell number, the density profiles derived from HLLC and HLLD are improved in the vicinity of *x* = 0.5 *m*, although the convergence rates are still very low due to their low-order nature. Relative CPU-times, which are the corresponding multiples relative to the CPU time taken by first order scheme coupled with HLL with the coarsest grid (N = 500), are given in Table 3 for different combinations of computational scheme and Riemann solver. While first order Godunov’s scheme is used, the CPU time spent by HLLC is approximately 1.7 times as long as that by HLL, and the time spent by HLLD is approximately 2.2 times as long as that by HLL. In summary, the first-order scheme can only give relatively reasonable solution even with very fine meshes and the use of HLLC and HLLD, having low convergence rate.

**Table 3:** Computation times for test case 2.

N	HLL	HLLC	HLLD	PPM + HLL	PPM + HLLC	PPM + HLLD
500	1.00	1.74	2.15	1.50	1.97	2.62
1000	4.25	6.80	8.52	5.26	7.80	11.27
2000	15.67	26.32	33.7	23.80	30.80	38.36

CPU times are shown relative to the first order scheme coupled with HLL Riemann solver on the coarsest grid.

**Figure 4:**
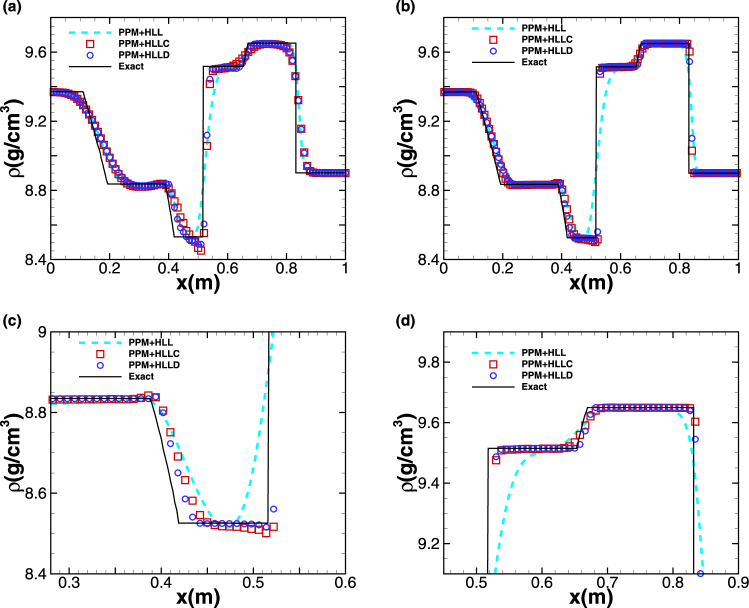
Five-wave shock-tube problem. The profiles of density at *t* = 0.06 *ms* computed by different schemes. The results computed using a mesh of (**a**) 100 cells. (**b**) 500 cells. (**c**,**d**) are partial enlargement of (**b**). The solid line represents the exact solution. In (**b**–**d**), the symbols are plotted with a spacing of 4 cells.

In the present framework, a simple way to increase the resolution of shock and discontinuity is to employ high-order reconstruction. Herein, we accomplish such reconstruction by applying Piecewise Parabolic Method (PPM), which is coupled with Riemann solvers, including HLL, HLLC and HLLD. Figure 4 plots the profiles of density derived from different combinations of PPM and Riemann solver: the one of PPM and HLL (denoted as ‘PPM + HLL’), the one of PPM and HLLC (denoted as ‘PPM + HLLC’) and the one of PPM and HLLD (denoted as ‘PPM + HLLD’). As shown in Fig. 4(a), the profiles can be resolved reasonably using a mesh containing only 100 cells. When the number of cells is increased to 500, the contact discontinuities, shock waves and the rarefaction wave in the region of 0.1 *m* ≤ *x* ≤ 0.2 *m* are all captured accurately, as shown in Fig. 4(b–d). Compared with exact solution, the scheme PPM + HLLD is seen to have the most accurate solution, particularly in the region of 0.4 *m* ≤ *x* ≤ 0.5 *m* where the solution exhibits very slow convergence rate. The CPU time spent by PPM + HLLC is approximately 1.3 times as long as that by PPM + HLL, while the time spent by PPM + HLLD is approximately 1.8 times as long as that by PPM + HLL. The *L*_1_-norm errors at time *t* = 0.06 *ms* and convergence rates for all combinations of PPM and Riemann solver are presented in Table 4 for test case 2.

**Table 4:** *L*_1_norm errors and orders of convergence for test case 2.

Method	N	*u*_1_		*u*_2_		*F*_11_	
		*L*_1_-error	*L*_1_-order	*L*_1_-error	*L*_1_-order	*L*_1_-error	*L*_1_-order
PPM + HLL	250	1.47E-02	—	2.51E-02	—	6.27E-03	—
	500	9.04E-03	0.704	1.70E-02	0.561	4.14E-03	0.599
	1000	5.24E-03	0.788	1.12E-02	0.600	2.77E-03	0.579
PPM + HLLC	250	8.85E-03	—	1.81E-02	—	2.82E-03	—
	500	5.13E-03	0.786	1.17E-02	0.633	1.77E-03	0.674
	1000	2.65E-03	0.955	7.19E-03	0.699	1.00E-03	0.819
PPM + HLLD	250	7.94E-03	—	9.65E-03	—	2.35E-03	—
	500	4.45E-03	0.835	5.76E-03	0.745	1.39E-03	0.761
	1000	2.11E-03	1.077	3.08E-03	0.904	7.07E-04	0.973

### Test case 3: Seven-wave shock-tube problem

Having assessed the performance of different schemes in the five-wave shock-tube example, we further test the accuracy and robustness of the high-order scheme PPM + HLLD in solving a more complex problem, i.e. the seven-wave shock-tube problem, by adding an additional degree of shear deformation. The initial conditions of this problem are

$$\begin{array}{l}{W}_{L}:\{u=(\begin{array}{c}0\\ 0.5\\ 1\end{array})\,km/s,F=(\begin{array}{ccc}0.98 & 0 & 0\\ 0.02 & 1 & 0.1\\ 0 & 0 & 1\end{array}),S={10}^{-3}\,kJ/gK\\ {W}_{R}:\{u=(\begin{array}{c}0\\ 0\\ 0\end{array})\,km/s,F=(\begin{array}{ccc}1 & 0 & 0\\ 0 & 1 & 0.1\\ 0 & 0 & 1\end{array}),S=0\,kJ/gK\end{array}$$

**Figure 5:**
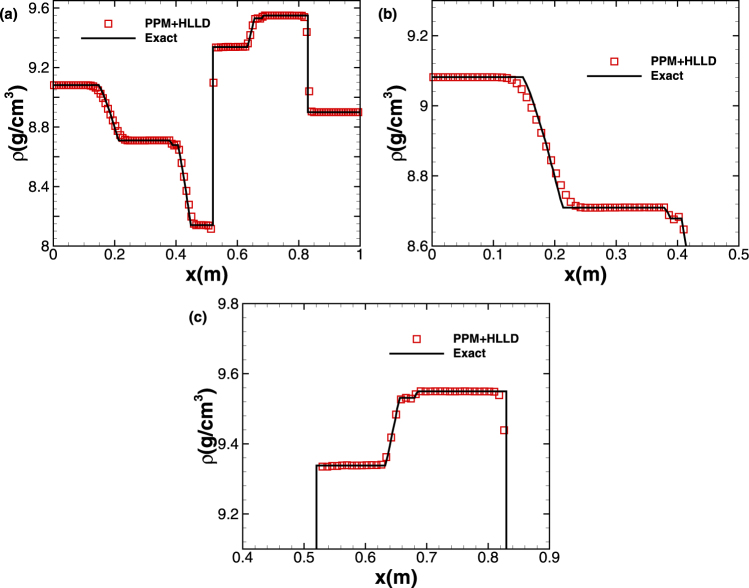
Seven-wave shock-tube problem computed by PPM coupled with HLLD. The profiles of density (**a**) at *t* = 0.06 *ms*. (**b**,**c**) are partial enlargement of (**a**). The symbols are plotted with a spacing of 4 cells.

The solution structure for this case is composed of three left-travelling rarefaction waves, a right-travelling contact discontinuity, two right-travelling rarefaction waves and a right-travelling shock wave. The results shown in Fig. 5 are obtained by the scheme PPM + HLLD on a mesh of 500 cells with the dimensional computational time *t* = 0.06 *ms*. As shown in Fig. 5, all the seven waves are captured precisely, implying that the PPM + HLLD scheme indeed has superiority and good robustness in processing the solid material problem with complex multi-wave structure. CPU times for test case 3 are comparable to those for test case 2 shown in Table 3. The *L*_1_-norm errors at time *t* = 0.06 *ms* and convergence rates of PPM + HLLD are presented in Table 5 for test case 3.

**Table 5:** *L*_1_ Errors and orders of convergence for the test case 3.

Scheme	N	*u*_1_		*u*_2_		*F*_11_	
		*L*_1_-error	*L*_1_-order	*L*_1_-error	*L*_1_-order	*L*_1_-error	*L*_1_-order
PPM + HLLD	250	7.20E-03	—	7.59E-03	—	2.63E-03	—
	500	3.75E-03	0.940	4.77E-03	0.669	1.53E-03	0.782
	1000	1.92E-03	0.966	2.59E-03	0.884	8.25E-04	0.892

### Test case 4: Solid/solid ‘stick’ problem

With the purpose of examining the performance of the method PPM + HLLD in the multi material problem, we will solve the solid/solid ‘stick’ problem and solid/solid ‘slip’ problem in the following two cases (test case 4 and 5) by using it. For the former, the materials at two sides of contact interface where the ‘stick’ condition is satisfied are the same and thus the Riemann solvers for both single-material and multi-material situations can be utilized for solution. When multi-material HLLD solver is used, level-set method for interface tracking must be applied in conjunction. This technique of PPM combined with multi-material HLLD solver and level-set method is referred to as ‘PPM + HLLD(M)’. And, the one of PPM coupled with single-material HLLD solver is denoted as ‘PPM + HLLD(S)’.

The initial conditions for test case 4 are

$$\begin{array}{l}{W}_{L}:\{u=(\begin{array}{c}2\\ 0\\ 0.1\end{array})\,km/s,F=(\begin{array}{ccc}1 & 0 & 0\\ -0.01 & 0.95 & 0.02\\ -0.015 & 0 & 0.9\end{array}),S=0\,kJ/gK\\ {W}_{R}:\{u=(\begin{array}{c}0\\ -0.03\\ -0.01\end{array})\,km/s,F=(\begin{array}{ccc}1 & 0 & 0\\ 0.015 & 0.95 & 0\\ -0.01 & 0 & 0.9\end{array}),S=0\,kJ/gK\end{array}$$

**Figure 6:**
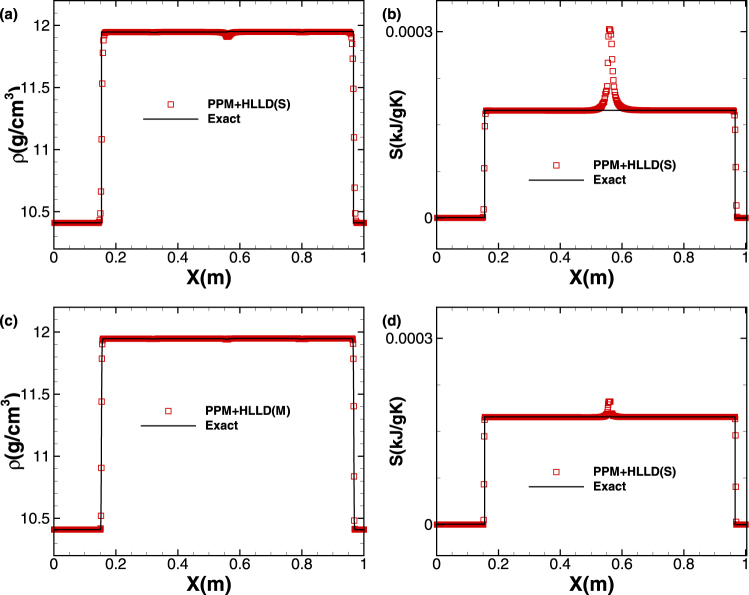
Solid/solid “stick” problem: profiles of density and entropy at *t* = 0.06 *ms*, which are computed by PPM + HLLD(S) and PPM + HLLD(M), respectively.

The solution structure comprises (from left to right): a left travelling longitudinal shock, transverse rarefaction wave, transverse shock, and the relevant right travelling waves, which are symmetric to the left travelling ones. In the solving process, the CFL number is set to be 0.6 and a mesh of 1000 cells is used in the computational domain. The profiles of density and entropy are shown in Fig. 6 at time *t* = 0.06 *ms*. Compared with the exact solution, the ones from PPM + HLLD(S) and PPM + HLLD(M) techniques are both featured by sharp peaks across shocks. Further, it is also found that spurious overshoots occur in both density and entropy profiles in the vicinity of *x* = 0.55, due to the fact that variables are not conserved across linearly degenerate field. Compared with PPM + HLLD(S), PPM + HLLD(M) can effectively suppress the spurious overshoot phenomenon due to the use of entropy fix technique, as illustrated in the Fig. 6(b,d). Nevertheless, it is noteworthy that this kind of error could not be eliminated completely in the present method framework, which still needs to be improved further in future.

*L*_1_ norm error and its convergence order for selected variables are shown in Table 6 with different cell numbers and method combinations such as 1st + HLLD, PPM + HLLD(S) and PPM + HLLD(M). It is found that *L*_1_ error norm for primitive variables exhibits approximately first-order convergence trend for both PPM + HLLD(S) and PPM + HLLD(M) techniques on account of the discontinuities (longitudinal shock, contact discontinuity and transverse shock) present in the solution. Further, the errors for all schemes decrease with increasing cell number, implying that the solutions are converging. For tangential velocity *u*_2_ and deformation gradient *F*_31_, the errors obtained by applying PPM on the coarsest mesh are lower than those by 1st-order Godunov’s scheme on the finest mesh, which indicates PPM indeed has higher order of reconstruction accuracy once again. In summary, the results for this case demonstrate that the material interface could be tracked accurately by level-set method, and highly precise solutions could be obtained by applying PPM + HLLD(M) with the spurious overshoots being suppressed simultaneously.

**Table 6:** *L*_1_ Errors and orders of convergence for the test case 4.

Scheme	N	*u*_1_		*u*_2_		*F*_31_	
		*L*_1_-error	*L*_1_-order	*L*_1_-error	*L*_1_-order	*L*_1_-error	*L*_1_-order
1st + HLLD	500	2.79E-03	—	1.15E-03	—	6.15E-04	—
	1000	1.37E-03	1.030	7.82E-04	0.551	4.21E-04	0.546
	2000	7.16E-04	0.931	5.37E-04	0.542	2.83E-04	0.572
PPM + HLLD(S)	500	2.74E-03	—	3.93E-04	—	1.23E-04	—
	1000	1.39E-03	0.983	2.35E-04	0.743	7.42E-05	0.725
	2000	6.77E-04	1.034	1.37E-04	0.781	4.15E-05	0.837
PPM + HLLD(M)	500	1.90E-03	—	3.04E-04	—	1.40E-04	—
	1000	1.00E-03	0.923	1.78E-04	0.768	7.73E-05	0.862
	2000	5.07E-04	0.987	9.73E-05	0.874	4.28E-05	0.853

### Test case 5: solid/solid ‘slip’ multi-material problem

**Figure 7:**
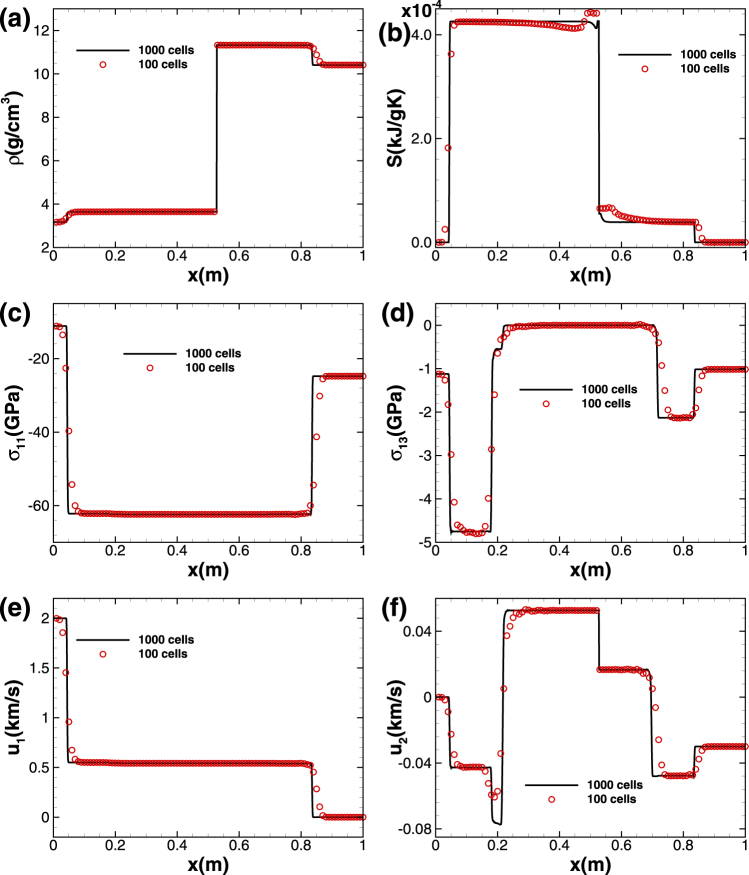
Solid/solid “slip” problem computed by the scheme PPM + HLLD(M). The profiles of density, entropy, stress components and velocities at t = 0.05 *ms*.

The initial conditions for this case are different from that for test case 5. In detail, the material at the left side of interface is now aluminum, while the one at the right side is still copper, with the slip condition satisfied at the material interface in the middle. The solution structure of this problem comprises six nonlinear waves, which are three left-travelling shocks inside the aluminum medium, three right-travelling shocks inside the copper medium, and a right-travelling contact discontinuity. We solve the problemm by means of the technique PPM + HLLD(M) for multi material situation within the dimensional time *t* = 0.05 *ms*. CFL number is fixed to be 0.6 and two meshes, including 100 cells and 1000 cells respectively, are used. Various variable profiles are shown in Fig. 7. With only 100 cells, the wave structure is captured reasonably by PPM + HLLD(M), although the numerical solution seems to be diffusive near the discontinuities. As the cell number is increased to 1000, the profiles of density, stress components and velocities near shocks become sharp without significant spurious oscillations, implying that PPM + HLLD(M) could be applied in multi material problems successfully. The *L*_1_ norm errors at time *t* = 0.05 *ms* and convergence rates of PPM + HLLD(M) are presented in Table 7 for test case 5.

**Table 7:** *L*_1_ Errors and orders of convergence for test case 5.

Scheme	N	*u*_1_		*u*_2_		*F*_11_	
		*L*_1_-error	*L*_1_-order	*L*_1_-error	*L*_1_-order	*L*_1_-error	*L*_1_-order
PPM + HLLD(M)	250	1.08E-02	—	1.91E-03	—	1.25E-03	—
	500	4.85E-03	1.155	9.41E-04	1.019	5.44E-04	1.197
	1000	2.26E-03	1.099	4.53E-04	1.055	2.56E-04	1.085

### Test case 6: impact of a projectile on a solid plate

**Figure 8:**
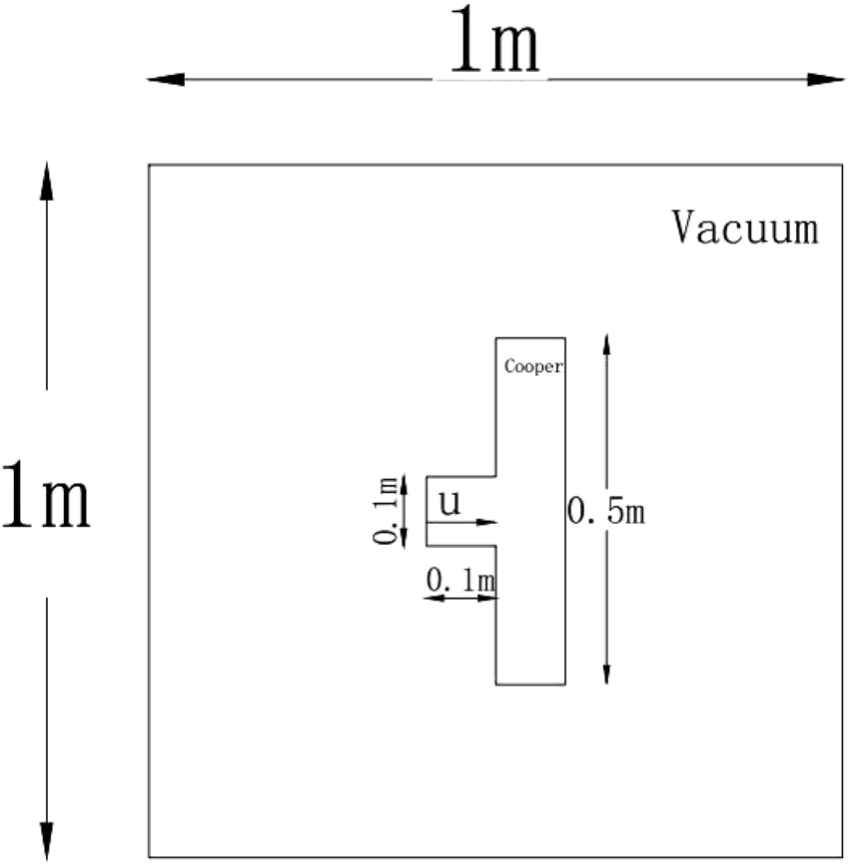
Schematic of initial conditions for the copper impact test.

In this two-dimensional case, the technique PPM + HLLD(M) for multi material situation is utilized to simulate the impact problem of a projectile on a solid plate surrounded by vacuum. The initial configuration is shown in Fig. 8. The projectile is a square with the length of 0.1 *m* while the plate is 0.5 *m* long and 0.1 *m* wide. The simulation starts from the moment when the projectile gets in touch with the plate. The materials of the projectile and plate considered are both copper. The computational grid covers the domain [−0.5 *m* ≤ *x* ≤ 0.5 *m*, −0.5 ≤ *y* ≤ 0.5 *m*] with uniform cell sizes in x and y directions as Δ*x* = Δ*y* = 1/1000 *m*, and the time step is determined from the CFL number which is fixed to be 0.6. At the initial time all materials are assumed to be in a stress free state: F = I and S = 0. Further, the cooper target is set to be static, while the copper projectile is initialized with a non-zero velocity component: *u*_1_ = 800 *m*/*s*. The solution of this test case was performed using a similar Eulerian model as that developed by Favrie^34^ and Gorsse^24^, where the projectile and plate are surrounded by air.

**Figure 9:**
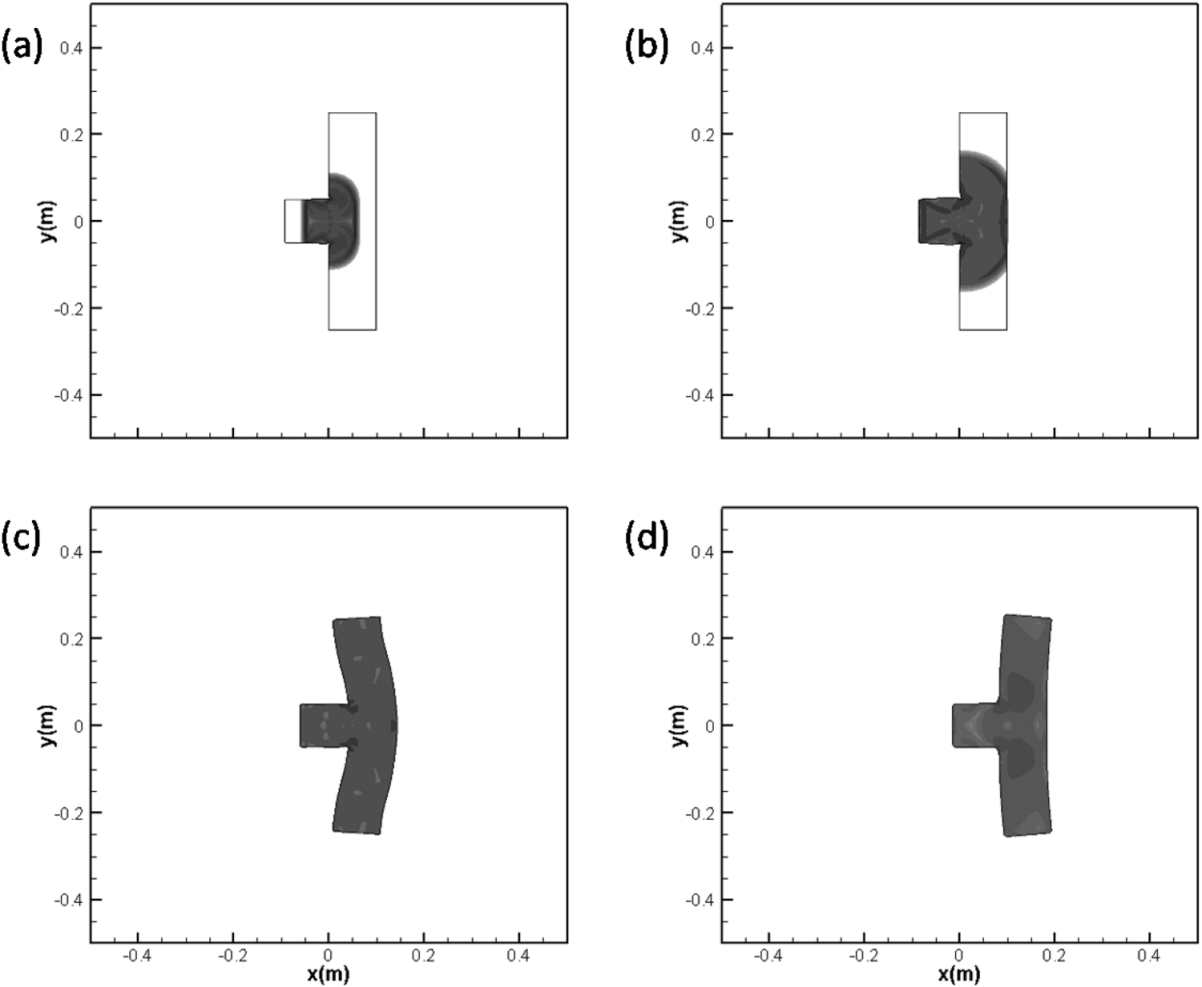
Impact at *u*_1_ = 800 *m*/*s* of a copper projectile on a copper plate at rest surrounded by vacuum. Numerical Schlieren pictures of the density at *t* = 1.0 × 10^−5^ *s*, *t* = 2.0 × 10^−5^ *s*, *t* = 2.3 × 10^−4^ *s* and *t* = 6.5 × 10^−4^ *s*.

The schlieren images of the density are shown at time *t* = 1.0 × 10^−5^ *s*, *t* = 2.0 × 10^−5^ *s*, *t* = 2.3 × 10^−4^ *s* and *t* = 6.5 × 10^−4^ *s* in Fig. 9. It is found that elastic shock propagates away from the impact surface, reaches the free surface and is subsequently reflected to form a rarefaction wave. Further, the elastic material is deformed and its surface oscillates with time, having strong similarity to the phenomena depicted by Favrie^34^ and Gorsse^24^. These facts may become one part of the evidence for the robustness of the technique PPM + HLLD(M) in two-dimensional cases.

### Test case 7: One-dimensional smooth problem

**Figure 10:**
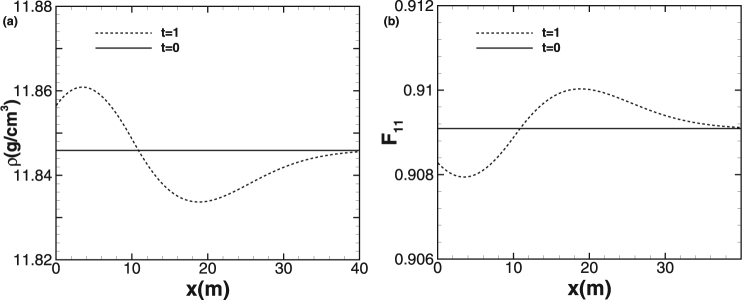
The initial conditions and computed results of the accuracy test problem using 400 cells. The profiles of density *ρ* (**a**) and deformation gradient *F*_11_ (**b**).

Finally, to test the accuracy of our method PPM + HLLD for smooth problems, we carry out the computation for a one-dimensional nontrivial and shockless problem with the material of copper and the initially Gaussian-shaped disturbance condition given by *F* = *I*/1.1, *u* = 0, *ε*_*i*_ = *ε*_1_ *ω*_*i*_ + (1 − *ω*_*i*_)*ε*_2_, *ε*_1_ = 0.823, *ε*_2_ = 10*ε*_1_, and ${\omega }_{i}=\mathrm{1/}a/\sqrt{2\pi }\,\exp (-{x}_{i}^{2}\mathrm{/(2}{a}^{2}))$, where *a* ^2^ = 100 is the distribution variance and *x*_*i*_ is the coordinate of the center of cell *i*. Further, the computational region is 0 *m* ≤ *x* ≤ 40 *m* and a transmissive boundary condition is used.The solution derived by high-order scheme with a fine mesh is utilized as the reference solution for accuracy and error analysis. The initial conditions and computed results at *t* = 1 *ms* are shown in Fig. 10 for this test case, for which the differences between the results from different grid resolutions are nearly invisible. The *L*_1_ norm errors for density *ρ*, normal velocity *u*_1_ and deformation gradient *F*_11_ at the output time *t* = 1 *ms* and the convergence rate of PPM + HLLD scheme are presented in Table 8, from which we could conclude that the results converge with the order of larger than 3, implying that PPM + HLLD is at least third-order accurate for smooth problems.

**Table 8:** *L*_1_ Errors and orders of convergence for test case 7.

Scheme	N	*ρ*		*u*_1_		*F*_11_	
		*L*_1_-error	*L*_1_-order	*L*_1_-error	*L*_1_-order	*L*_1_-error	*L*_1_-order
PPM + HLLD	100	5.41E-04	—	3.41E-04	—	4.44E-04	—
	200	5.37E-05	3.334	3.55E-05	3.265	4.08E-05	3.444
	400	5.10E-06	3.396	3.56E-06	3.320	3.90E-06	3.386

## Discussion

In this study, the governing equations developed by Godunov & Romenski^14,31^ are utilized to describe the elastic deformation of solid materials in Eulerian reference frame. The existing Godunov-type shock-capturing schemes have been applied in conjunction with HLL family of Riemann solver to solve Riemann problems in nonlinear elasticity. The two-state HLL Riemann solver^7^ has been widely used as the standard shock-capturing scheme due to its simplicity and high effectiveness. However, numerical experiments show that HLL solver is of strong dissipation particularly in the cases with contact surfaces and strong shock waves, leading to inaccurate resolution of physical features and unacceptable numerical smearing. HLLC^35,36^ method assumes a three-wave model and thus it could lead to better resolution of intermediate waves. Nevertheless, for systems with the eigenstructures containing more than three distinct characteristic fields, HLLC seems to be inadequate and tends to behave like HLL with inaccurate resolution of intermediate waves, particularly when these waves move slowly relative to the mesh used, as illustrated in test case 1. Therefrom, HLLC solver has been improved and developed to be HLLD solver by admitting the correct number of characteristic field^37,38^. HLLD solver involves five wave structure and has been successfully applied in MHD^39,40^. In this paper we extend the usage of HLLD solver to the impact problem of nonlinear elasticity, considering the similarity of the wave structures in nonlinear elasticity with those appearing in MHD. And, a comparative study shows that 1st-order Godunov’s scheme coupled with HLLD solver has advantages in capturing multi-wave structures in solid material with large deformation, in comparison with that coupled with HLL and HLLC solvers.

With the purpose of increasing the convergence rate of solution, we apply the well-known piecewise parabolic method, an extension of 1st-order Godunov’s scheme to higher order accuracy, and couple it with HLL-type Riemann solvers to solve the problems featured with complex nonlinear wave structures. The results of numerical experiments show that the technique PPM + HLLD provides solutions with highest resolution for single material problems compared with other techniques. Moreover, as HLLD Riemann solver can deal with boundary conditions at the interface between different solid materials, we develop the technique PPM + HLLD(M) by coupling PPM with multi-material HLLD Riemann solver for the treatment of multi material problems, where level-set algorithm is used for tracking material interfaces. As shown in the solid/solid ‘stick’ problem, the PPM + HLLD(M) technique in conjunction with level-set algorithm can suppress greatly ‘overshoot’ or ‘heating errors’, which may occur in the vicinity of contact discontinuity as observed in the work of Titarev^20^. For the solid/solid ‘slip’ multi-material problem, it is demonstrated that the PPM + HLLD(M) technique is able to capture nonlinear wave structure accurately even in the presence of strong shocks. While for the two-dimensional impact problem (test case 6), the PPM + HLLD(M) technique can reproduce similar dynamic behaviors with those given by Favrie^34^ and Gorsse^24^, proving the robustness of this technique in two-dimensional cases. Further, compared with the method adopted by Barton *et al*.^30^ on the basis of characteristic relation of invariants, the PPM + HLLD technique is relatively easier to be implemented in the existing code. The accuracy analysis on our PPM scheme confirms that it exhibits approximately third-order convergence and even achieves fourth-order accuracy with time step Δ*t* tending to 0 for smooth problem. The relevant results of accuracy and error analysis imply that for the Riemann problem for elasticity with discontinuity, linearly degenerate discontinuous waves produce sub-linear convergence rates which eventually dominate the global convergence rates. In our examples, the convergence rates with first-order Godunov’s scheme are close to 0.5, while those with PPM are close to 0.75, being consistent with the theoretical rates for contact discontinuities presented by J.W. Banks^41^, where the authors reported that for a general scheme of order *p*, the order of convergence for unlimited stable schemes is established as *p*/(*p* + 1). In summary, the PPM + HLLD technique has been proved to be a robust tool in solving Riemann problems in nonlinear elasticity for both single material problem and multi material problem, and it could be natural to be extended to plastic problems.

## Methods

### Governing equations of nonlinear elasticity

The model developed by Godunov & Romenski^14,31^ is used to describe the deformation process of solid material in the Eulerian reference frame. The physical variables used contain velocities *u*_*i*_, deformation gradient tensor *F*_*ij*_ = ∂*x*_*i*_/∂*X*_*j*_ (where *x*_*i*_ and *X*_*j*_ denote the fixed Eulerian coordinates and Lagrangian coordinates respectively) and specific entropy *S*. Following the notations used by Titarev^20^ and Barton^21^, a hyperbolic equation system depicting momentum, strain, and energy conservation laws in Cartesian coordinates can be written as

1 $$\frac{\partial \rho {u}_{i}}{\partial t}+\frac{\partial (\rho {u}_{i}{u}_{k}-{\sigma }_{ik})}{\partial {x}_{k}}=0$$

2 $$\frac{\partial \rho {F}_{ij}}{\partial t}+\frac{\partial (\rho {F}_{ij}{u}_{k}-\rho {F}_{kj}{u}_{i})}{\partial {x}_{k}}=0$$

3 $$\frac{\partial \rho E}{\partial t}+\frac{\partial (\rho {u}_{k}{\rm{E}}-{u}_{i}{\sigma }_{ik})}{\partial {x}_{k}}=0$$

Here *ρ* denotes the material density, *σ* is the Cauchy stress, *E* = *ε* + *u*_*i*_ *u*_*i*_/2 is the total energy, *ε* is the specific internal energy and the Einstein summation convention over repeated indices is implied (*i*, *j*, *k* = 1, 2, 3). The material density, stress tensor, specific internal energy, strain tensor *G*_*ij*_ and temperature *T* can be represented as functions of the variables mentioned above:

4 $$\rho ={\rho }_{0}/{\rm{\det }}|{\bf{F}}|,\,{\sigma }_{ij}=\rho {F}_{ik}\frac{\partial \varepsilon }{\partial {F}_{jk}}=-2\rho {G}_{ik}\frac{\partial \varepsilon }{\partial {G}_{jk}},\,\varepsilon =\varepsilon ({F}_{ij},S),\,T=\frac{\partial \varepsilon }{\partial S},\,G={{\bf{F}}}^{-T}{{\bf{F}}}^{-1}$$

where *ρ*_0_ is the density of the initially unstressed medium. Further, the continuity equation is expressed as

5 $$\frac{\partial \rho }{\partial t}+\frac{\partial \rho {u}_{k}}{\partial {x}_{k}}=0$$

Following the treatment by Barton^42^, we also use the continuity equation (5) to replace the strain conversation equation for the deformation gradient component *ρF*_11_ for convenience. Thereafter, the governing equations can be expressed in matrix form:

6 $$\frac{\partial {\bf{U}}}{\partial t}+\frac{\partial {{\bf{F}}}^{i}}{\partial {x}_{i}}=-{{\bf{S}}}_{C}$$

where **U** is the vector composed of the conservative variables, **F** ^*i*^ is the corresponding flux vector and **S**_*C*_ is the vector of source terms associated with the constraints for the deformation tensor:

7 $${\bf{U}}=(\begin{array}{c}\rho {\bf{u}}\\ \rho \\ \rho {F}_{12}\\ \rho {F}_{13}\\ \vdots \\ \rho {F}_{33}\\ \rho {\rm{E}}\end{array}),\,{{\bf{F}}}^{i}=(\begin{array}{c}{u}_{i}\rho {\bf{u}}-\sigma {e}_{i}\\ \rho {u}_{i}\\ {u}_{i}\rho {F}_{12}-{u}_{1}\rho {F}_{i2}\\ {u}_{i}\rho {F}_{13}-{u}_{1}\rho {F}_{i3}\\ \vdots \\ {u}_{i}\rho {F}_{33}-{u}_{3}\rho {F}_{i3}\\ {u}_{i}\rho E-(\sigma u){e}_{i}\end{array}),\,{{\bf{S}}}_{C}=(\begin{array}{c}0\\ 0\\ {u}_{1}\frac{\partial \rho {F}_{j2}}{\partial {x}_{j}}\\ {u}_{1}\frac{\partial \rho {F}_{j3}}{\partial {x}_{j}}\\ \vdots \\ {u}_{3}\frac{\partial \rho {F}_{j3}}{\partial {x}_{j}}\\ 0\end{array})$$

Introducing the vector of primitive variables **W** = (**u**, *F* ^*T*^ *e*_1_, *F* ^*T*^ *e*_2_, *F* ^*T*^ *e*_3_, *S*), we could rewrite Equation (6) as a hyperbolic quasi-linear system

8 $$\frac{\partial {\bf{W}}}{\partial t}+{{\bf{A}}}^{k}\frac{\partial {\bf{W}}}{\partial {x}_{k}}=0$$

where the Jacobian matrix **A** ^*k*^ is given by

9 $${{\bf{A}}}^{k}=[\begin{array}{ccccc}{u}_{k}{\rm{I}} & -{A}^{k1} & -{A}^{k2} & -{A}^{k3} & -{B}^{k}\\ -{F}^{T}{D}_{k1} & {u}_{k}{\rm{I}} & 0 & 0 & 0\\ -{F}^{T}{D}_{k2} & 0 & {u}_{k}{\rm{I}} & 0 & 0\\ -{F}^{T}{D}_{k3} & 0 & 0 & {u}_{k}{\rm{I}} & 0\\ 0 & 0 & 0 & 0 & {u}_{k}\end{array}]$$

Here, ${A}_{ij}^{lm}=\frac{1}{\rho }\frac{\partial {\sigma }_{li}}{\partial {F}_{mj}}$, ${B}_{i}^{l}=\frac{1}{\rho }\frac{\partial {\sigma }_{li}}{\partial S}$ and ${D}_{ij}={e}_{i}\otimes {e}_{j}^{T}$. See Barton^21^ for details on the eigenstructure of this system.

The equation of state (EOS) for elastic solid employed is the formula about the specific internal energy *ε*, which is expressed in terms of three independent invariants of the Finger tensor (*I*_1_, *I*_2_, *I*_3_)^20,43^:

10 $$\varepsilon =\frac{{K}_{0}}{2{\alpha }^{2}}{({I}_{3}^{\alpha \mathrm{/2}}-\mathrm{1)}}^{2}+{c}_{v}{T}_{0}{I}_{3}^{\gamma \mathrm{/2}}({e}^{S/{c}_{v}}-1)+\frac{{B}_{0}}{2}{I}_{3}^{\beta \mathrm{/2}}({I}_{1}^{2}\mathrm{/3}-{I}_{2})$$

where

11 $${I}_{1}=tr\,{\bf{G}}={G}_{11}+{G}_{22}+{G}_{33},\,{I}_{2}=\frac{1}{2}[(tr\,{\bf{G}}{)}^{2}-tr({{\bf{G}}}^{2})],\,{I}_{3}=det\,{\bf{G}},$$

${K}_{0}={c}_{0}^{2}-\mathrm{(4/3)}{b}_{0}^{2}$ and ${B}_{0}={b}_{0}^{2}$ are the squared speeds of the pressure and shear waves, respectively. *c*_*v*_ is the heat capacity at constant volume, and *T*_0_ is the reference temperature. Further, *α*, *β* and *γ* are all constants characterizing the non-linearities in the EOS^20^.

### Finite Volume Methods

Considering now the one-dimensional system of elastic solid and taking *k* = 1 in Equation (6), the following conservative equation is obtained:

12 $$\frac{\partial {\bf{U}}}{\partial t}+\frac{\partial {\bf{F}}}{\partial x}=0,$$

where the source term **S**_*c*_ becomes equal to zero and is neglected in numerical computation for one-dimensional cases. Note that for two-dimensional and three-dimensional cases, the source item **S**_*c*_ is not neglected and treated as source terms in the numerical computation for the purpose of capturing the correct wave speed in the quasi-linear system (Equation (8)) and ensuring the correctness of numerical results. Equation (6) is not conservative in nature for multi-dimensional case. Nevertheless, we follow standard practice^21,22^ and solve the two-dimensional impact problem (test case 6) by using Equation (6) sequentially. With grid spacing Δ*x* = *x*_*i*+1/2_ − *x*_*i*−1/2_ and time step Δ*t* = *t* ^*n*+1^ − *t* ^*n*^, the finite volume scheme for solving this hyperbolic Equation (12) can be written as

13 $${{\bf{U}}}_{i}^{n+1}={{\bf{U}}}_{i}^{n}-\frac{{\rm{\Delta }}t}{{\rm{\Delta }}x}({{\bf{F}}}_{i+\mathrm{1/2}}-{{\bf{F}}}_{i-\mathrm{1/2}})$$

where ${{\bf{U}}}_{i}^{n}$ is an approximation to the average of spatial integral in the cell [*x*_*i*+1/2_, *x*_*i*−1/2_] and **F**_*i*+1/2_ is the numerical flux yet to be defined. As proposed by Godunov^16^, the numerical flux **F**_*i*+1/2_ is derived by solving the Riemann problem with the initial data

14 $${\bf{U}}(x,\,\mathrm{0)}=\{\begin{array}{ll}{{\bf{U}}}_{L}={{\bf{U}}}_{i}^{n}, & x < 0\\ {{\bf{U}}}_{R}={{\bf{U}}}_{i+1}^{n}, & x > 0\end{array}$$

In this study, we use HLL (Harten, Lax and van Leer) family of solvers to obtain approximate solutions of the Riemann problem in nonlinear elasticity and carry out a comparative study on their robustness and accuracy. It is worthwhile that for HLL-type solvers, the wave propagation speed (*λ*_*k*_) and the wave decomposition of Δ*q* into Δ_*k*_ *q* are not rigorously derived from the Jacobian matrix, leading to their usability for simulating the problems with complicated Jacobian matrix. In the following, we will briefly introduce HLLD Riemann solver. While to see the details of HLL and HLLC Riemann solver, please refer to Harten^7^, Gavrilyuk^4^ and Toro^15^.

#### HLLD Riemann Solver

**Figure 11:**
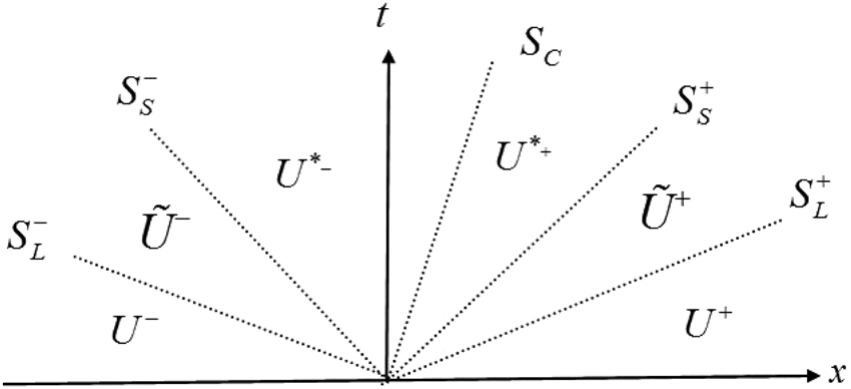
HLLD Riemann solver.

HLLD Riemann solver gives a nonlinear approximate solution. Its central idea is to assume a wave configuration for the solution that consists of five waves (two slow waves, two fast waves and a contact discontinuity), which separates six constant states. As shown in Fig. 11, there are four intermediate states: ${\tilde{{\bf{U}}}}^{-}$, **U** ^*−^, **U** ^*+^, and ${\tilde{{\bf{U}}}}^{+}$. The fastest (longitudinal) waves between **U** ^±^ and ${\tilde{{\bf{U}}}}^{\pm }$ are denoted ${S}_{L}^{\pm }$ and the slow shear waves ${S}_{S}^{\pm }$ separate the states ${\tilde{{\bf{U}}}}^{\pm }$ and **U** ^*±^. Each wave is considered to be a discontinuity and the Rankine-Hugoniot relation is satisfied across each wave (${S}_{S}^{\pm }$ and ${S}_{L}^{\pm }$):

15a $${S}_{L}^{\pm }{\tilde{{\bf{U}}}}^{\pm }-{\tilde{{\bf{F}}}}^{\pm }={S}_{L}^{\pm }{{\bf{U}}}^{\pm }-{{\bf{F}}}^{\pm }={{\bf{Q}}}_{L}^{\pm }$$

15b $${S}_{S}^{\pm }{\tilde{{\bf{U}}}}^{\pm }-{\tilde{{\bf{F}}}}^{\pm }={S}_{S}^{\pm }{{\bf{U}}}^{\ast \pm }-{{\bf{F}}}^{\ast \pm }={{\bf{Q}}}_{S}^{\pm }$$

15c $${S}_{C}{{\bf{U}}}^{\ast -}-{{\bf{F}}}^{\ast -}={S}_{C}{{\bf{U}}}^{\ast +}-{{\bf{F}}}^{\ast +}$$

From Eqs (15a–15c) one can find that there are more unknowns than equations, thus some other conditions must be imposed. In order to obtain the unknown intermediate state vectors ${\tilde{{\bf{U}}}}^{\pm }$, ${{\bf{U}}}^{\ast \pm }$, ${\tilde{{\bf{F}}}}^{\pm }$ and ${{\bf{F}}}^{\ast \pm }$, the following conditions need to be satisfied:

- Tangential velocities *u*_2_, *u*_3_ and tangential stresses *σ*_12_, *σ*_13_ are continuous across fast (longitudinal) waves and may jump across slow (shear) waves.
- Density *ρ*, normal velocity *u*_1_ and normal stress *σ*_11_ are continuous across slow waves and may jump across fast waves.
- Normal stress *σ*_11_ and normal velocity *u*_1_ are continuous across contact discontinuity; for the ‘stick’ multi-material problem, shear stress and tangential velocity are equal for different materials at the interface; while for the ‘slip’ multi-material problem, tangential component of the stress vector are zero.

The left and right fastest wave speeds are approximated from the information of original state by

16 $${S}_{L}^{-}=\,{\rm{\min }}({\lambda }_{1}({{\bf{W}}}_{L}),{\lambda }_{1}({{\bf{W}}}_{R}));\,{S}_{L}^{+}=\,{\rm{\min }}({\lambda }_{13}({{\bf{W}}}_{L}),{\lambda }_{13}({{\bf{W}}}_{R}))$$

Once the states ${\tilde{{\bf{U}}}}^{\pm }$ are known, the slow wave speeds are estimated by

17 $${S}_{S}^{-}=\,{\rm{\min }}({\lambda }_{2}({\tilde{{\bf{W}}}}_{L}),{\lambda }_{2}({\tilde{{\bf{W}}}}_{R}));\,{S}_{S}^{+}=\,{\rm{\min }}({\lambda }_{12}({\tilde{{\bf{W}}}}_{L}),{\lambda }_{12}({\tilde{{\bf{W}}}}_{R}))$$

Under HLL approximation^15^, the intermediate wave speed *S*_*C*_ in the present solver is evaluated as

18 $$\begin{array}{rcl}{S}_{C} & = & \frac{({S}^{+}{\rho }^{+}{u}_{1}^{+}-{S}^{-}{\rho }^{-}{u}_{1}^{-})-({\rho }^{+}{u}_{1}^{+}{u}_{1}^{+}-{\sigma }_{11}^{+}-({\rho }^{-}{u}_{1}^{-}{u}_{1}^{-}-{\sigma }_{11}^{-}))}{({S}^{+}{\rho }^{+}-{S}^{-}{\rho }^{-})-({\rho }^{+}{u}_{1}^{+}-{\rho }^{-}{u}_{1}^{-})}\\  & = & \frac{{Q}_{L}^{+}\mathrm{(1)}-{Q}_{L}^{-}\mathrm{(1)}}{{Q}_{L}^{+}\mathrm{(4)}-{Q}_{L}^{-}\mathrm{(4)}}\end{array}$$

where ${Q}_{L}^{\pm }(i)$ denotes the *i*-th component of the vector ${Q}_{L}^{\pm }$ (the meaning of ${Q}_{R}^{\pm }(i)$ can be inferred accordingly). From these wave speeds above, $\tilde{{\bf{U}}}$ state can be obtained as:

19 $$\begin{array}{rcl}{\tilde{u}}_{1}^{\pm } & = & {S}_{C};\,{\tilde{u}}_{\mathrm{2,3}}^{\pm }={u}_{\mathrm{2,3}}^{\pm }\mathrm{;\ \ \ }\\ {\tilde{\rho }}^{\pm } & = & {\rho }^{\pm }\frac{{S}_{L}^{\pm }-{u}_{1}^{\pm }}{{S}_{L}^{\pm }-{S}_{C}}=\frac{{Q}_{L}^{\pm }\mathrm{(4)}}{{S}_{L}^{\pm }-{S}_{C}};\\ {\tilde{\sigma }}_{11}^{\pm } & = & {\rho }^{\pm }({S}_{L}^{\pm }-{u}_{1}^{\pm })\,({u}_{1}^{\pm }-{S}_{C})+{\sigma }_{11}^{\pm };\,{\tilde{\sigma }}_{12}^{\pm }={\sigma }_{12}^{\pm };\,{\tilde{\sigma }}_{13}^{\pm }={\sigma }_{13}^{\pm };\\ {\tilde{F}}_{1i}^{\pm } & = & ({\rho }^{\pm }{F}_{1i}^{\pm })/{\tilde{\rho }}^{\pm };\,{\tilde{F}}_{2i}^{\pm }={F}_{2i}^{\pm };\,{\tilde{F}}_{3i}^{\pm }={F}_{3i}^{\pm };\\ {\tilde{E}}^{\pm } & = & ({Q}_{L}^{\pm }\mathrm{(13)}-{{\tilde{u}}_{i}}^{\pm }{\tilde{\sigma }}_{1i}^{\pm })/{Q}_{L}^{\pm }\mathrm{(4).}\end{array}$$

And, the intermediate states ${{\bf{U}}}^{\ast }$ are constructed as:

20 $${\rho }^{\ast \pm }={\tilde{\rho }}^{\pm };\,{u}_{1}^{\ast \pm }={\tilde{u}}_{1}^{\pm };\,{\sigma }_{11}^{\ast \pm }={\tilde{\sigma }}_{11}^{\pm }$$

For the ‘stick’ multi-material problem, the following formulas are established:

21 $$\begin{array}{rcl}{u}_{2}^{\ast +} & = & {u}_{2}^{\ast -}=\frac{{\sigma }_{12}^{-}-{\sigma }_{12}^{+}+{\tilde{\rho }}^{-}{u}_{2}^{-}({S}_{S}^{-}-{S}_{C})-{\tilde{\rho }}^{+}{u}_{2}^{+}({S}_{S}^{+}-{S}_{C})}{{\tilde{\rho }}^{-}({S}_{S}^{-}-{S}_{C})-{\tilde{\rho }}^{+}({S}_{S}^{+}-{S}_{C})}=\frac{{Q}_{S}^{+}\mathrm{(2)}-{Q}_{S}^{-}\mathrm{(2)}}{{Q}_{S}^{+}\mathrm{(4)}-{Q}_{S}^{-}\mathrm{(4)}};\\ {u}_{3}^{\ast +} & = & {u}_{3}^{\ast -}=\frac{{\sigma }_{13}^{-}-{\sigma }_{13}^{+}+{\tilde{\rho }}^{-}{u}_{3}^{-}({S}_{S}^{-}-{S}_{C})-{\tilde{\rho }}^{+}{u}_{3}^{+}({S}_{S}^{+}-{S}_{C})}{{\tilde{\rho }}^{-}({S}_{S}^{-}-{S}_{C})-{\tilde{\rho }}^{+}({S}_{S}^{+}-{S}_{C})}=\frac{{Q}_{S}^{+}\mathrm{(3)}-{Q}_{S}^{-}\mathrm{(3)}}{{Q}_{S}^{+}\mathrm{(4)}-{Q}_{S}^{-}\mathrm{(4)}};\\ {\sigma }_{12}^{\ast \pm } & = & {Q}_{S}^{\pm }\mathrm{(2)}-{u}_{2}^{\ast \pm }{Q}_{S}^{\pm }\mathrm{(4);}\\ {\sigma }_{13}^{\ast \pm } & = & {Q}_{S}^{\pm }\mathrm{(3)}-{u}_{3}^{\ast \pm }{Q}_{S}^{\pm }\mathrm{(4).}\end{array}$$

While for the ‘slip’ multi-material problem, one can obtain

22 $${u}_{2}^{\ast \pm }={u}_{2}^{\pm }+{\sigma }_{12}^{\pm }/{Q}_{S}^{\pm }\mathrm{(4);}\,{u}_{3}^{\ast \pm }={u}_{3}^{\pm }+{\sigma }_{13}^{\pm }/{Q}_{S}^{\pm }\mathrm{(4);}\,{\sigma }_{12}^{\ast \pm }={\sigma }_{13}^{\ast \pm }=0$$

and expressions of other variables are given as follows

23 $$\begin{array}{l}{F}_{1i}^{\ast \pm }={\tilde{F}}_{1i}^{\pm };\,{F}_{21}^{\ast \pm }=({Q}_{S}^{\pm }\mathrm{(7)}-{(\rho {F}_{11}{u}_{2})}^{\ast \pm })/{Q}_{S}^{\pm }\mathrm{(4);}\\ {F}_{22}^{\ast \pm }=({Q}_{S}^{\pm }\mathrm{(8)}-{(\rho {F}_{12}{u}_{2})}^{\ast \pm })/{Q}_{S}^{\pm }\mathrm{(4);}\,{F}_{23}^{\ast \pm }=({Q}_{S}^{\pm }\mathrm{(9)}-{(\rho {F}_{13}{u}_{2})}^{\ast \pm })/{Q}_{S}^{\pm }\mathrm{(4);}\\ {F}_{31}^{\ast \pm }=({Q}_{S}^{\pm }\mathrm{(10)}-{(\rho {F}_{11}{u}_{3})}^{\ast \pm })/{Q}_{S}^{\pm }\mathrm{(4);}\,{F}_{32}^{\ast \pm }=({Q}_{S}^{\pm }\mathrm{(11)}-{(\rho {F}_{12}{u}_{3})}^{\ast \pm })/{Q}_{S}^{\pm }\mathrm{(4);}\\ {F}_{33}^{\ast \pm }=({Q}_{S}^{\pm }\mathrm{(12)}-{(\rho {F}_{13}{u}_{3})}^{\ast \pm })/{Q}_{S}^{\pm }\mathrm{(4);}\,{E}^{\ast \pm }=({Q}_{S}^{\pm }\mathrm{(13)}-{{u}_{i}}^{\ast \pm }{\sigma }_{1i}^{\ast \pm })/{Q}_{S}^{\pm }\mathrm{(4)}\end{array}$$

Within a single material, the boundary condition at the interface is set to be ‘stick’, and the HLLD fluxes ${\tilde{{\bf{F}}}}^{\pm }$ and ${{\bf{F}}}^{\ast \pm }$ for Godunov’s scheme are then given by

24 $${{\bf{F}}}_{hlld}=\{\begin{array}{ll}{{\bf{F}}}^{-} & if\,{S}_{L}^{-} > 0,\\ {\tilde{{\bf{F}}}}^{-} & if\,{S}_{L}^{-}\le 0\le {S}_{S}^{-},\\ {{\bf{F}}}^{\ast -} & if\,{S}_{S}^{-}\le 0\le {S}_{C},\\ {{\bf{F}}}^{\ast +} & if\,{S}_{C}\le 0\le {S}_{S}^{+},\\ {\tilde{{\bf{F}}}}^{+} & if\,{S}_{S}^{+}\le 0\le {S}_{L}^{+},\\ {{\bf{F}}}^{+} & if\,{S}_{L}^{+} < 0.\end{array}$$

### Extension to Multi-material Problem

#### Interface Evolution of Level-Set Equation

The level-set algorithm is used in the simulation of multi material problem for interface tracking. The moving interface Γ(*t*) is the zero isosurface of level-set function *φ*(**x**, *t*) at any moment and *φ*(**x**, 0) represents the signed distance from point **x** to interface Γ(0).

25 $$\phi ({\bf{x}},\,0)=\{\begin{array}{ll}d({\bf{x}},{\rm{\Gamma }}(0)), & {\bf{x}}\in {{\rm{\Omega }}}^{1},\\ \mathrm{0,} & {\bf{x}}\in {\rm{\Gamma }}(0),\\ -d({\bf{x}},{\rm{\Gamma }}(0)), & {\bf{x}}\in {{\rm{\Omega }}}^{2},\end{array}$$

where *d*(*φ*, Γ(0)) is the unsigned distance from **x** to Γ(0).

The ideal method of contour surface function is that *φ* moves at an appropriate speed. At any moment, as long as the value of *φ* is fixed, we could determine the position of moving interface, based on which the governing equations are spontaneous to be solved.

*φ* should satisfy certain governing equation. At any moment *t*, *φ*(**x**, *t*) equals to zero for any point **x** on moving interface Γ(*t*), leading to the following equation:

26 $$\frac{d\phi }{dt}=\frac{\partial \phi }{\partial t}+\frac{d{\bf{x}}}{dt}\cdot {\rm{\Delta }}\phi =0,\quad V=\frac{d{\bf{x}}}{dt}$$

In general, with increasing time, *φ*(**x**, *t*) will not satisfy signed distance any longer. In order to maintain its property, reinitialization algorithm, which transforms *φ*(**x**, *t*) to make it be the signed distance from point **x** to interface Γ(*t*), is adopted. The transformation is realized by obtaining stable solutions of the initial value problem:

27 $$\{\begin{array}{l}{\phi }_{\tau }={\rm{sign}}({\phi }_{0})(1-|\nabla \phi |),\\ \phi ({\bf{x}}\mathrm{,0})={\phi }_{0}.\end{array}$$

Spatial derivatives in Equation (26) are discretised using 5th-order weighted essentially non-oscillatory scheme (WENO) and time integration is performed using 3rd-order Runge-Kutta scheme.

#### HLLD Multi-Material Riemann Solver

For multi-material problems, the solving procedure is similar to HLLD Riemann solver in single material problem except that ‘stick’ or ‘slip’ boundary conditions should be considered at the material interface. For an interface located between the grid points *i* and *i* + 1, the known states **W**_*L*_ = **W**(*x*_*i*_, *t* ^*n*^) and **W**_*R*_ = **W**(*x*_*i*+1_, *t* ^*n*^) at the current time level, corresponding to the left and right materials respectively, are used to pose a multi-material Riemann problem. The fluxes at the left and right sides are given as

28 $${{\bf{F}}}_{i+1/2}^{l}={{\bf{F}}}^{\ast -},{{\bf{F}}}_{i+1/2}^{r}={{\bf{F}}}^{\ast +}$$

The states in the respective materials’ ghost cells are defined as **W**_*i*+1_ = **W**_*i*+2_ = **W**_*i*+3_ = **W** ^*−^ and **W**_*i*−2_ = **W**_*i*−1_ = **W**_*i*_ = **W** ^*+^. Meanwhile, an entropy fix technique is adopted to suppress ‘heating errors’ (see Liu *et al*.^44^ for the details of the technique). In practical applications, the initial values of **W**_*L*_ and **W**_*R*_ are set to be **W** ^*−^ and **W** ^*+^ respectively and utilized to conduct multiple iterations. The quantities computed will converge after about 10 iterations.

### Data availability statement

The datasets generated and/or analysed during the current study are available from the corresponding author on reasonable request.
